# Comparison of quantitative laser speckle contrast and indocyanine green imaging for intestinal perfusion measurements in robot assisted surgery

**DOI:** 10.1038/s41598-025-05496-x

**Published:** 2025-07-01

**Authors:** M. M. Coraci, H. M. Schouw, S. Kruijff, Y. Mao, M. E. Noltes, W. Heeman

**Affiliations:** 1 Department of Surgery, University Medical Centre Groningen, Groningen, The Netherlands; 2 Department of Nuclear Medicine and Molecular Imaging, University Medical Centre Groningen, Groningen, The Netherlands; 3 Department of Molecular Medicine and Surgery, Karolinska Institute, Stockholm, Sweden; 4 ©Ronovo Surgical Inc, Shanghai, China; 5 Department of Surgery, Martini Hospital, Groningen, The Netherlands; 6 LIMIS development B.V, Leeuwarden, The Netherlands

**Keywords:** Preclinical research, Imaging and sensing

## Abstract

Adequate perfusion is essential to prevent anastomotic leakage in intestinal and rectal surgeries. This study compares Laser Speckle Contrast Imaging (LSCI) and Indocyanine Green Fluorescence Angiography (ICG-FA) for assessing blood flow in intestinal anastomoses during robot-assisted surgeries in pigs. Intestinal perfusion was evaluated in three pigs using LSCI and ICG-FA, before and after clamping the main arterial supply, with measurements taken across ten regions of interest (ROIs). Pearson correlation coefficients were used to compare the two techniques. ROIs were normalized for analysis to facilitate direct comparison of the perfusion patterns. The results showed a strong correlation between the maximum fluorescence intensity from ICG-FA and LSCI values after clamping (*r* = 0.7293), with comparable perfusion patterns observed post-unclamping. LSCI provides continuous monitoring, while ICG-FA captures contrast-enhanced snapshots, explaining weaker correlations for static values. No significant difference was found in normalized measurements between the two methods. This study supports the use of both LSCI and ICG-FA in clinical practice, highlighting their complementary roles in assessing perfusion during surgeries. Further research is needed to explore their combined utility.

## Introduction

Anastomotic leakage (AL) is a frequent and severe complication following colorectal surgery, resulting from the failure of the anastomosed ends to heal and fuse. The complete aetiology of AL is still unclear due to its multifactorial origin, with inadequate blood flow serving as a common underlying mechanism that contributes to or exacerbates many of the diverse causes^1^. The incidence of AL can vary significantly depending on the anatomical site of the anastomosis, ranging up to 19%, with colorectal or coloanal anastomoses showing the highest incidence^2^. Although surgeons routinely evaluate signs of adequate blood supply like tissue colour, a palpable pulse within the supplying mesentery, and bleeding edges of transected ends, studies show that these methods of assessment lack predictive accuracy for AL^3^. As such, a more standardized and reproducible method of intraoperative assessment of anastomotic perfusion may alter surgical strategy in a significant number of patients and lower AL rates^4^.

Indocyanine green fluorescence angiography (ICG-FA) is an imaging technique that leverages the fluorescent properties of indocyanine green (ICG) when exposed to light at near-infrared wavelengths (NIR)^5^. Upon intravenous administration, ICG binds to plasma proteins and remains confined within the vascular system^5^. In gastrointestinal surgery, ICG-FA is increasingly used to evaluate anastomotic perfusion with the aim of reducing the incidence of AL. While some studies could not demonstrate benefits of using ICG^6^several meta-analyses comparing the difference in the incidence of AL after colorectal surgery between qualitative ICG-FA assessment and standard treatment found that the use of ICG-FA was linked to a reduction in AL^7–9^. Despite its growing use, the evidence supporting ICG-FA effectiveness in significantly lowering AL rates remains inconclusive and a quantitative assessment of ICG-FA data is not yet carried out in practice^10,11^. The use of ICG-FA is not without limitations. The technique requires the injection of an exogenous dye, and its utility in repeated assessments is constrained by the pharmacokinetics of ICG. Furthermore, standardization and quantification are required for objective decision making but are considered challenging^12^. The major advantage of ICG-FA is that it can identify in and outflow of feeding and draining vessels.

Laser Speckle Contrast Imaging (LSCI) is another optical perfusion imaging technique that generates 2-D perfusion images of large tissue surfaces. LSCI works by illuminating tissue with coherent laser light, which creates a random interference pattern known as a speckle pattern. When red blood cells in vessels move, they cause fluctuations in this pattern, leading to blurring in the speckle images. This blurring is directly related to blood flow, thus allowing LSCI to visualize flow in real-time without the need for a fluorescent dye. The advantage of LSCI over ICG-FA is that it can provide multiple and continuous assessments of microvascular perfusion during surgery as it does not require a contrast agent. Furthermore, LSCI provides a more precise measure of the flow compared to ICG, which innately measures the blood volume^13^. LSCI has been applied in various clinical settings, including imaging burn wounds, retinal perfusion, cerebral blood flow, and skin microvasculature, though its use in routine clinical practice remains limited to date^14^. Thus far, some studies have compared ICG-FA and LSCI qualitatively and quantitatively^13,15–21^. These studies looked at factors such as usability of both methods, the correlation of the speckle pattern to visible ICG scores in human parathyroid glands and the correlation of quantitative ICG peak inflow slopes to laser speckle perfusion units (LSPU)^17,18,21^. Despite the established use of LSCI and ICG-FA, no study has yet compared their effectiveness within the same field of view during robot-assisted surgery. Imaging in the same field allows for real-time perfusion comparisons, minimizing the risk of spatial variations affecting the measurements. This study aims to quantitatively evaluate the comparability of ICG-FA and LSCI during robot-assisted small intestine and rectal surgeries using a porcine model in the same field of view, enhancing our understanding of their respective roles in perfusion assessment.

## Methods

### Surgical procedure

**Fig. 1:**
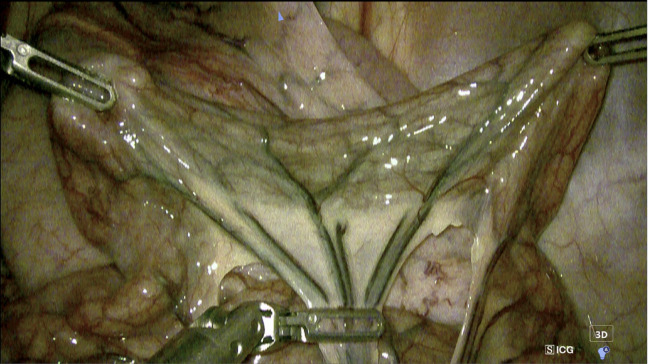
The intestinal segment held by the Carina™ robot system. Two arms (upper left and right) are used to lift the intestinal tissue. A third arm (center bottom) is used to clamp the arteries.

All procedures involving pigs were conducted following an IACUC-approved protocol (protocol #PLJC23-224) and in compliance with applicable ethical guidelines, laws, and regulations, including ARRIVE guidelines. Three experimental pigs were sourced from Jurong Kangrong Poultry Industry Co., Ltd. (Jiangsu, China). Anaesthesia was induced using atropine and Zoletil™ and maintained with isoflurane. Once anaesthesia was achieved, a pneumoperitoneum was created, and three 8 mm robotic ports along with a 5 mm assistant port were inserted. The robot was then docked to the lower abdominal region according to the specific procedural setup. Following docking, the peritoneum was incised to expose the gastrointestinal tract. Two of the robotic arms were utilized to stabilize a segment of the small intestine, approximately ± 15 centimetres. To create an ischemic segment, a third robotic arm was employed to clamp the feeding peripheral arteries supplying the corresponding intestinal segment (Fig. 1). LSCI and ICG-FA images were acquired from the rectal and small intestinal segments, following the imaging procedure described below. After completing imaging with both modalities, the experiment was concluded. The animals were then euthanized under general anaesthesia by administering 1 mL/kg saturated potassium chloride (KCl).

### Carina™ robotic assisted surgery system

The Carina™ platform, developed by the Chinese company ©Ronovo Surgical Inc., is a MMPA approved robotic-assisted surgery platform designed for laparoscopic procedures across multiple specialties. It consists of a surgeon console and multiple patient-side carts with robotic arms. The modular architecture allows for flexible configuration based on the surgical needs.

### ICG-FA system

The Storz IMAGE1 S™ Rubina^®^ (Tuttlingen, Germany) 3D fluorescence imaging system was used for the ICG-FA. The system utilizes a class 1 laser as the excitation light source and a near-infrared sensitive camera for video capture. The camera was set to ICG mode, and all settings were kept the same during each experiment. ICG (Zhuhai Ruidu Pharmaceutical Co., Ltd., China) was injected as a dilution of 25 mg dye in 10 mL saline solution, followed by a 2 ml saline flush to promote rapid entry of ICG into the blood stream. The raw fluorescence data was saved on a laptop using a frame grabber. The ICG data was later quantified using ImageJ.

### PerfusiX-Imaging™

PerfusiX-Imaging™ developed by Dutch company LIMIS development B.V (Leeuwarden, The Netherlands), a novel dye free method for intraoperative perfusion assessment, was used in this study. The system is designed to be integrated with existing laparoscopic video systems, such as in this case the Storz Rubina™ system, providing real-time visualization of microperfusion during minimally invasive or robot assisted surgery. The PerfusiX-Imaging™ was added to the video tower of the Carina™ robotic system and connected to the Storz 3D Rubina Vision™ system such as has been described before^22^. The device uses red laser light to homogenously illuminate the tissue of interest. The raw speckle images were captured using a frame grabber and saved for further analysis. The data was analysed using proprietary quantification software. The colormap indicates high flow (yellow) and low flow (blue), based on laser speckle perfusion units (LSPU). The quantification software allowed for the placement of regions of interest (ROI’s) on the intestinal tissues.

### Small intestine LSCI and ICG-FA perfusion assessment

**Fig. 2:**
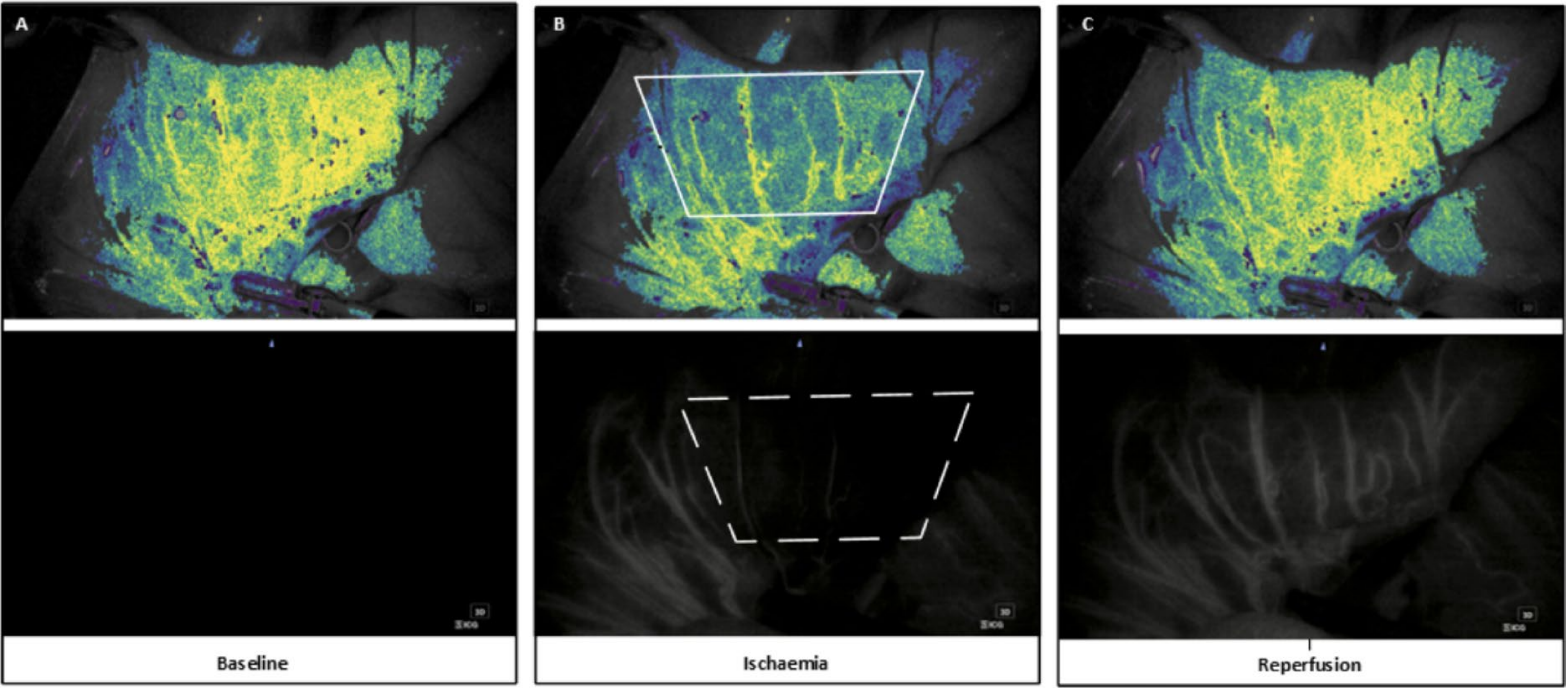
Images produced at different phases of image acquisition using LSCI (top) and ICG-FA (bottom). A baseline was recorded for 10 s (**A**), for LSCI before artery clamping and for ICG with the artery clamped. Ischemia was then introduced for a period of 30 s by clamping the feeding artery (**B**), Lastly, reperfusion was recorded for the following 30 s by releasing the clamped artery (**C**).

Assessment of perfusion with LSCI was done using PerfusiX-Imaging™ using the time intervals of no clamp (10 s), clamp (30 s), no clamp (30 s). Subsequently, the peripheral arteries were re-clamped in preparation for the ICG data recording. An ICG bolus (2 mg/kg) was administered, and data collection using the ICG camera system was started at the moment of dye injection. ICG images were collected, with fixed settings, of the following time intervals: clamp (30 s) and no clamp (30 s). Figure 2 illustrates the three phases of image acquisition using both imaging modalities.

### Rectal ICG-FA perfusion assessment

After a minimum waiting period of 90 min, a similar methodology was applied to measure the rectal ischemia reperfusion of the same pig. The waiting period ensured complete clearance of ICG from the pig. The main feeding artery (superior rectal artery) was clamped to induce ischemia. First, images were obtained with LSCI using the following intervals; no clamp (10 s) – clamp (30 s) - no clamp (30 s). Subsequently ICG was injected (bolus 2 mg/kg) followed by the saline flush. ICG-FA was performed using the following intervals clamp (30 s) – no clamp (30 s).

### Image processing

**Fig. 3:**
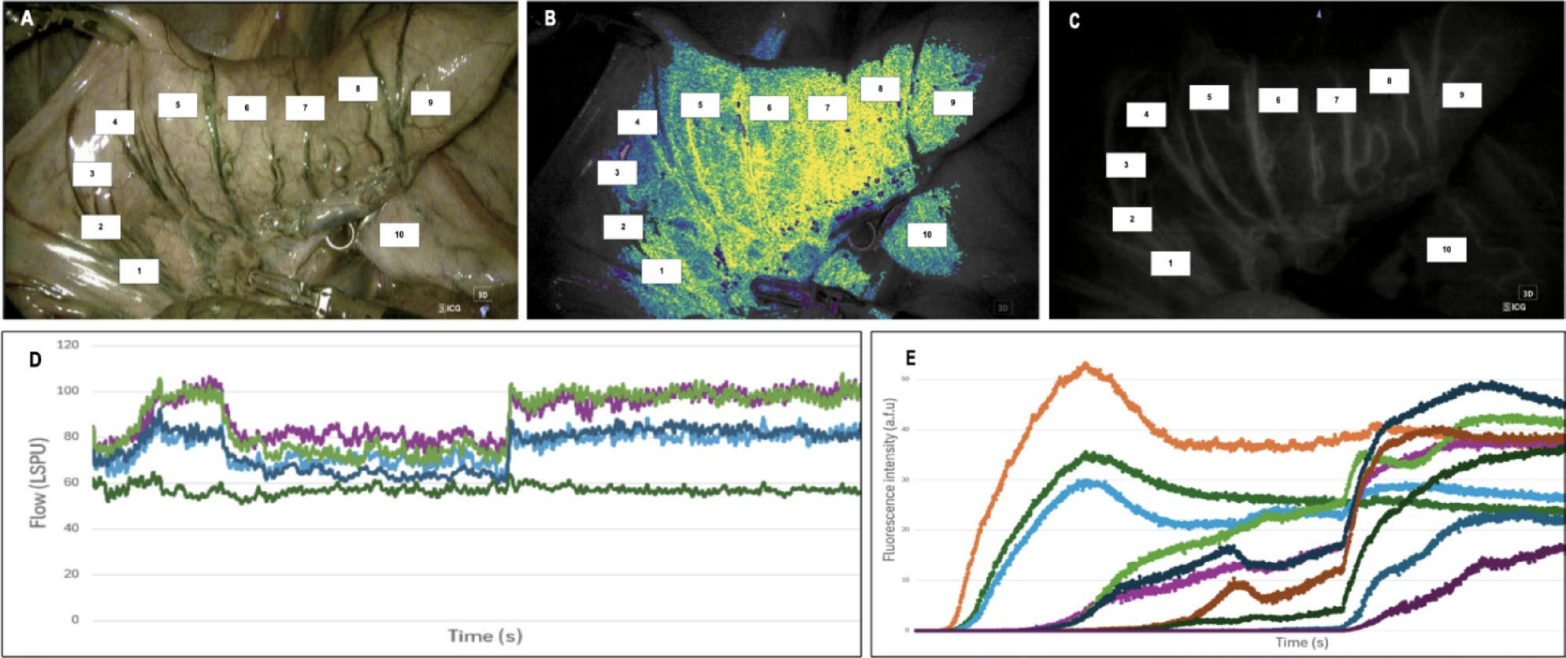
Assessment setup. The rectum segment was suspended using robotic arms (A) The segments were assessed with LSCI (B) and ICG-FA(C). ROIs were chosen from left to right along the intestine segment (1–10). D, The LSCI curve for this segment. Different colours represent different ROIs. E, The ICG-FA fluorescence intensity curve for this segment, where different colours represent different ROI.

**Fig. 4:**
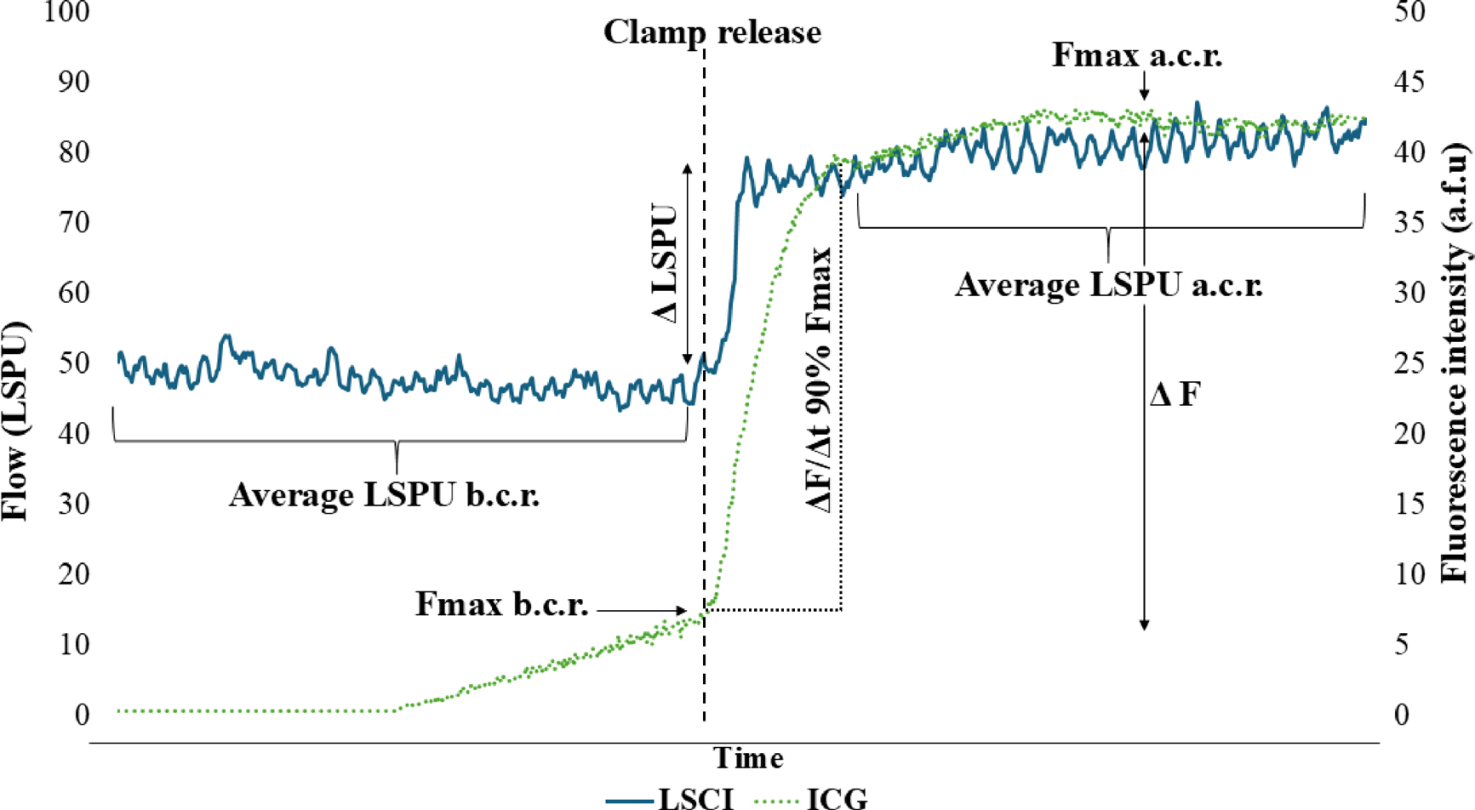
Example of extracted laser speckle contrast imaging (LSCI) and indocyanine green fluorescence angiography (ICG-FA) curves. The following parameters can be extracted from the ICG-FA curves: Max fluorescence intensity (Fmax) measured in a.f.u., and ICG-FA slope to 90% of Fmax value (ΔF/Δt 90%). For LSCI, laser speckle pattern analysis, measured in laser speckle perfusion units (LSPU) were extracted. Parameters can be measured before clamp release (b.c.r) and after clamp release (a.c.r).

For each LSCI data set, ten regions of interest (ROI) were drawn from left to right, avoiding main arteries as these regions show a high signal intensity (Fig. 3). The LSCI signal was extracted with PerfusiX-Imaging™ research software. Graphs of the LSCI signal over time were plotted (Fig. 4).

The same ten ROI’s were analysed for ICG-FA (Fig. 3). The ICG-FA movie files were converted to image stacks. The image stacks were analysed using the ImageJ software. The fluorescence intensity was analysed over time, resulting in the corresponding perfusion curves (Fig. 4). The clamp/unclamp time point was determined by looking at the white light images and comparing these with LSCI and ICG perfusion curves. The parameters extracted from the perfusion curves before and after unclamping are listed in Table 1.

**Table 1:** Parameters extracted from LSCI and ICG-FA perfusion curves.

LSCI	ICG-FA
Average before unclamping (LSPU),	Maximum fluorescence intensity per ROI (Fmax) before unclamping (a.f.u.).
Average after unclamping (LSPU)	Maximum fluorescence intensity per ROI (Fmax) after unclamping (a.f.u.)
Delta LSPU (increase in LSPU after unclamping) (LSPU)	Delta fluorescence intensity (increase in fluorescence intensity after unclamping) (a.f.u.)
*(Inflow slope cannot be calculated for LSCI)*	Inflow slope till 90% of the Fmax value (slope 90%max) (a.f.u./t)

### Data analysis

Pearson correlations coefficients were determined between several LSCI and ICG-FA parameters according to Table 2. Furthermore, for each ROI, the Fmax and maximum LSCI values before and after clamping were normalized to the highest value in each measurement (per pig, per tissue type). Subsequently, all normalized data of all pigs and tissue types was pooled per ROI and the average was calculated. A Shapiro-Wilk test was performed to determine whether the data was normally distributed, and a paired t-test to test whether there was a significant difference in perfusion measurement between the two modalities. The normalized average Fmax and maximum LSCI values across all ROIs (1–10) from both intestinal and rectal measurements were plotted to compare the relative change in blood flow of LSCI and ICG-FA.

## Results

In three porcine specimens, both intestinal and rectal perfusion were imaged using LSCI and ICG-FA. For each image series, 10 ROIs were analysed, resulting in a total of 60 data points. All collected data was included in the study.

### Correlations

**Fig. 5:**
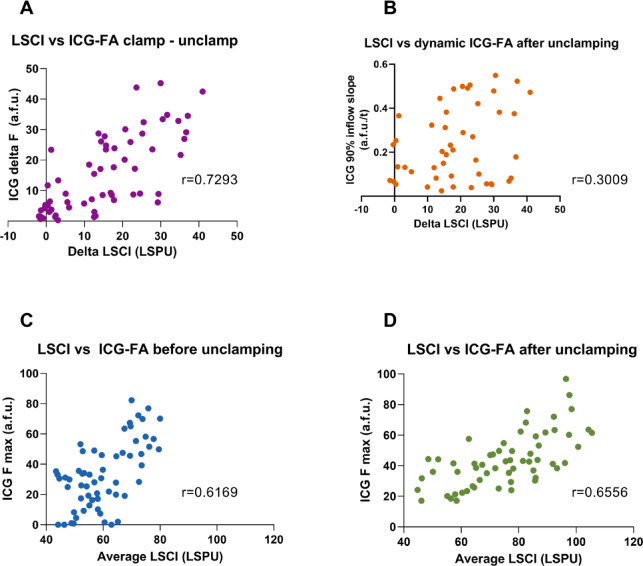
Plots of Pearson correlations between several laser speckle contrast imaging (LSCI) and Indocyanine green perfusion angiography (ICG-FA) parameters. ICG delta Fmax correlates strongly to Delta LSCI (*r* = 0.7293**) (A)**, ICG 90% inflow slope correlates weakly to the delta LSCI value (*r* = 0.3009) **(B)**, ICG Fmax and Average LSCI before and after unclamping show a moderate correlation (*r* = 0.6169 and *r* = 0.6556 respectively (**C** and **D**).

The results of the Pearson correlation between the various perfusion parameters of both imaging modalities are depicted in Table 2; Fig. 5. The change in Fmax before and after clamping (delta Fmax) correlates strongly to the change in LSCI values (delta LSCI) (*r* = 0.7293) (Fig. 5A). Additionally, the Fmax values before unclamping showed a moderate correlation with the corresponding maximum LSPU value before unclamping (*r* = 0.6169) (Fig. 5C). This was also observed for the corresponding Fmax and LSPU values after unclamping (*r* = 0.6556) (Fig. 5D). The inflow slope however only has a weak to moderate correlation to the delta LSCI (*r* = 0.3009) (Fig. 5B).

**Table 2:** Correlated ICG-FA and LSCI parameters and their corresponding r value and strength of correlation.

Correlation	*r* value	Strength
ICG delta F vs. delta LSCI	0.7293	Strong
ICG 90% inflow slope vs. delta LSCI	0.3009	Moderate
ICG Fmax vs. average LSCI before unclamping	0.6169	Moderate
ICG Fmax vs. average LSCI after unclamping	0.6556	Moderate

### Spatial analysis

**Fig. 6:**
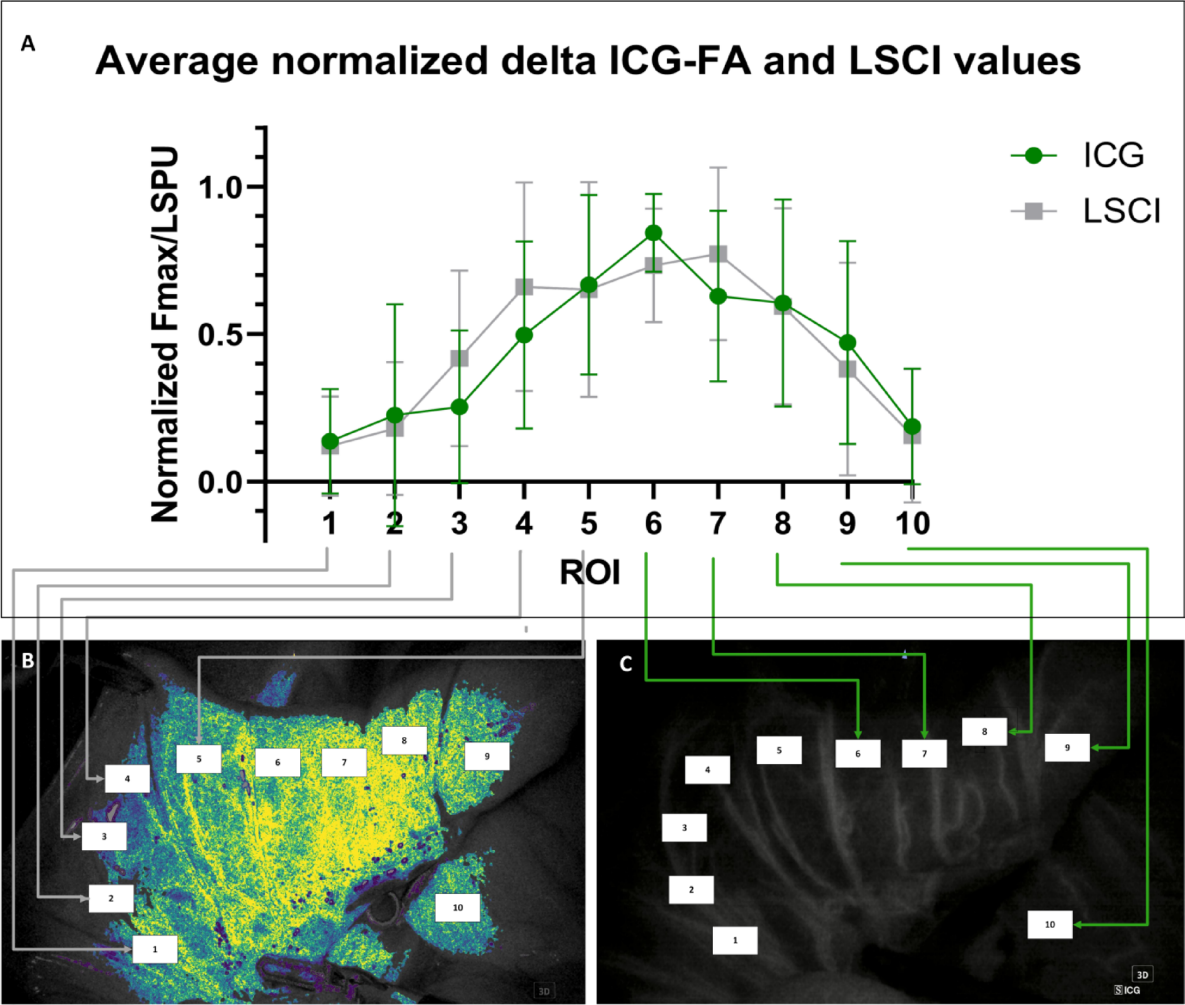
Average normalized delta ICG and delta LSPU values with standard deviation per ROI for both intestinal and rectal perfusion measurements (A). The grey arrows from A to B and the green arrows to C illustrate that each point on the graph represents an ROI (only 5 arrows were drawn towards the LSCI and ICG-FA images respectively, however all 10 ROI for both imaging modalities appear in graph A).

To examine how ICG-FA and LSCI compare in measuring flow per ROI, the delta Fmax and delta LSCI were normalized to the ROI with the highest value within each measurement (Pig 1–3, intestine and rectum). The Shapiro-Wilk test revealed normal distribution of the data (*p* = 0.181 and 0.464 for LSCI and ICG-FA respectively) and there is no significant difference between the LSCI and ICG-FA data when a paired t-test was performed (*p*=−0.657). The average normalized values per ROI were plotted in order from left to right. Figure 6 illustrates that the average delta Fmax and delta LSPU were highest in the ROIs close to middle of the tissue specimen where the clamp was placed (ischemia) and released (reperfusion). This aligns with the qualitatively observed area of ischaemia resulting from clamping the artery supplying the central part of the specimen followed by reperfusion upon release of the clamp, resulting in a high delta Fmax and delta LSPU values. Moreover, the lower delta values towards the lower and higher ROIs can be attributed to the fact that these areas, located at the edge of the specimen, are unaffected by the central clamping of the arteries and thus maintain a constant state of perfusion.

## Discussion

This study is the first to directly compare LSCI and ICG-FA in the evaluation of intestinal and rectal perfusion during robot-assisted surgery. The integration of the laser speckle perfusion imaging in the robot assisted surgery platform Carina™ was successful. Although not all extracted parameters correlate, both imaging modalities effectively visualize ischemic areas and reflect perfusion changes in a quantitative manner. However, ICG is limited in capturing dynamic blood flow alterations after dye injection.

Notably, our results demonstrate that both modalities can detect changes in perfusion over time, with strong agreement between delta LSCI and delta Fmax values representing pre- and post-unclamping differences. This study found that (snapshot) values such as average LSCI and Fmax before or after unclamping show a moderate correlation. This discrepancy can possibly be explained by the fundamental differences in the principles underlying LSCI and ICG-FA. LSCI measures blood flow by assessing the movement of red blood cells, while ICG-FA detects the presence of fluorescent dye. As a result, there can be situations where LSCI detects no flow due to the absence of detectable movement, while ICG-FA still indicates the presence of fluorescence, potentially due to stagnant residual dye in the tissue thus leading to a false positive. This difference in measurement mechanisms may lead to instances where LSCI and ICG-FA yield different results, particularly in low-flow or complex flow conditions. Our findings indicate that ICG-FA and LSCI can comparably measure changes in flow resulting from arterial unclamping. However, significant differences, advantages, and limitations exist between these modalities. LSCI, for instance, is highly sensitive to motion artifacts, however, the PerfusiX-Imaging™ device’s proprietary algorithms can correct for this^18^. In this study, the use of the Carina™ robot provided notable benefits, particularly in stabilizing tissue and ensuring a steady camera platform. Additionally, the use of the robot ensured a stable field of view and yielded consistent and reproducible images for both LSCI and ICG-FA, highlighting the advantages of robotic assisted surgery platforms in perfusion imaging. However, many robotic fluorescence imaging systems are limited by the fact that that they implement auto-scaling or lack the ability to extract raw imaging data, complicating accurate quantification and objective measurement, both of which are essential for linking perfusion to function.

Also, in general, ICG-FA provides only a snapshot of perfusion, lacking the ability to continuously monitor perfusion changes. Repeated or continuous measurements are difficult because the dye remains in the bloodstream for some time. As such, a limitation of ICG-FA, unlike LSCI, is that it cannot measure a baseline followed shortly by a decrease in perfusion, as the dye must first be cleared before a new measurement can be made. Nonetheless, ICG-FA can visualize outflow, illustrating the rate at which the fluorescent signal decreases after reaching its maximum (Fmax) (see Fig. 3E, orange line). LSCI lacks this capability, limiting its ability to discern between inflow and outflow dynamics^17,23–28^. Yet, LSCI provides the possibility for multiple and longer measurements since it is not dependent on the presence or clearance of exogenous dyes.

There are several other dye-free optical perfusion imaging techniques besides LSCI, each with distinct advantages and limitations. Hyperspectral imaging, for example, can visualize blood oxygenation and deoxygenation, but the signal quality can be compromised by ambient lighting, leading to a low signal-to-noise ratio. Additionally, hyperspectral imaging does not provide real-time data, data analysis is complex, and its equipment is not yet compatible with laparoscopic imaging systems^29,30^.

However, to determine whether ICG-FA and LSCI can complement each other in clinical practice, it is crucial to further elucidate the relationship between LSPU- and ICG-derived metrics in future clinical trials. For example, both our study and others have reported no significant correlation between average or maximum LSPU and the maximum slope of ICG, indicating that while these modalities are both effective in measuring perfusion, the specific parameters they assess may not align directly^31^. This consistent observation underscores the need for more in-depth studies to better understand how these measurements relate to each other. Future studies are also needed to compare the capabilities of LSCI and ICG-FA in measuring or predicting clinical consequences of quantitative perfusion measurements.

Our findings align with existing literature, such as from Zötterman et al., who used a Pearson rank correlation to compare average perfusion (LSCI) and area under the curve (ICG-FA) in the assessment of tissue flaps^19^. They found a moderate correlation between these parameters (*r* = 0.55, *P* < 0.0001). In line with our findings, their study also reported no significant correlation between LSPU values and the maximum inflow slope of ICG (*r* = 0.11, *P* = 0.18)^19,31^. Other studies provided a more descriptive analysis of LSCI and ICG-FA in gastrointestinal perfusion assessment, reporting that blood flow boundaries matched in 65% of cases and that both modalities can significantly distinguish between perfused and ischemic regions with comparable accuracy^20,21^. Noël et al. also compared ICG-FA and LSCI by assessing renal perfusion in a porcine model. The perfusion boundary between the clamped and unclamped upper segmental artery was similarly visualized by both imaging modalities. This finding is concurrent with our study, however, our study also enabled quantitative assessment^32^. Additionally, to our knowledge, our study is the first to utilize imaging within the same field of view, enabling real-time perfusion comparisons and minimizing the risk of temporal variations affecting this comparison.

The current study has a few limitations. First, the experiments were conducted on only three pigs. As we did not see any unreasonable outliers, we do expect similar results in a larger experiment. Hence, to respect the current directives on animal experimentation containing the 3R’s (replacement, reduction and refinement), the current study design was deemed sufficient to study our current aims^33^. Despite attempts in previously published literature to compare these modalities, there is no widely accepted method for directly comparing LSCI and ICG-FA. This paper’s data interpretation reflects our approach to the challenge of comparing both modalities. Lastly, the Storz ICG mode can perform automatic adjustments, and the lack of a researcher mode prevents us from ensuring that no automatic alterations were made during imaging. However, the fact that the camera was fixated by the robot minimizes the risk of drastic alterations influencing the measurements.

This study provides a quantitative comparison between ICG-FA and LSCI, demonstrating that both modalities similarly measure dynamic perfusion changes. However, static or snapshot values, do not correlate as well likely due to fundamental differences in their underlying measurement principles. These differences may lead to discrepancies between LSCI and ICG-FA, particularly in low-flow or complex flow conditions such as a false positive due to the presence of stagnant fluorescent dye. The use of a robot assisted surgery platform enabled the exact same field-of-view for both modalities allowing direct comparison. The added stability of a robotic arm also seems beneficial for the stability of laser speckle perfusion measurements and laser speckle perfusion visualization. The method developed here for comparing these techniques contributes to the growing field of intraoperative optical perfusion imaging. Although limited by a small sample size, our findings suggest that LSCI is comparable to ICG-FA for dynamic perfusion changes. Further research is required to explore whether these modalities can be interchanged or are complementary to enhance tissue perfusion clinical decision-making during surgery.

## Data Availability

Original datasets generated during and/or analyzed during the study are available from the corresponding author on reasonable request.
